# Data on mechanical and thermal properties of an amine-epoxy system at various post-curing temperatures

**DOI:** 10.1016/j.dib.2025.112109

**Published:** 2025-09-25

**Authors:** Alex Tamayo-Aguilar, Víctor H. Guerrero, Patricia I. Pontón, Marco V. Guamán

**Affiliations:** a Department of Materials, Escuela Politécnica Nacional, Quito 170109, Ecuador; b Department of Mechanical Engineering, Escuela Politécnica Nacional, Quito 170109, Ecuador

**Keywords:** Thermosets, Post-curing, Dynamic mechanical analysis, Flexural test, Thermal analysis

## Abstract

The aim of this work is to provide a manufacture guide for producing a tailor-made amine-epoxy system that can operate at different services temperatures depending on the post-curing treatment. Therefore, a data set compilation of the mechanical and thermal properties of a commercial thermoset system (Epikure 3223 amine/Epon 828 epoxy) was obtained after exposure to a room temperature cure followed by different post-cures (80, 100, 120 °C), all below the fully cured glass transition temperature (T_g_∞ ∼ 153 °C). The mechanical properties of this epoxy system were determined through dynamic mechanical analysis (DMA) and flexural testing, while its thermal behavior was evaluated by differential scanning calorimetry (DSC), thermomechanical analysis (TMA), and thermogravimetric analysis (TGA). The data was analyzed using analysis of variance (ANOVA) to assess if the selected post-curing temperatures provoke a statistically significant difference on the corresponding properties. This data highlights the importance of selecting an adequate post-curing process to extend the temperature range of the glassy state, ensuring reproducible mechanical and thermal properties up to temperatures close to T_g_∞, which is key for high temperature applications in automotive, aeronautic, aerospace and construction. Furthermore, this approach guarantees that the curing kinetics would no longer influence the thermal history of this epoxy system.

Specifications Table

**Table:** 

Subject	Engineering & Materials science
Specific subject area	Mechanical and thermal characterization of structural thermosets.
Type of data	Table, Figure. Raw, Analyzed.
Data collection	DSC: TA Instruments Q2000 analyzer using TA Instruments Universal software version 4.5, in accordance with the procedure indicated in the ASTM E2602–22 standard. DMA: TA Instruments Discovery DMA 850 analyzer, using TRIOS software version 5.0 in compliance with ASTM D5418 and ASTM E1640–18 standards. TMA: TA Instruments Q400 analyzer using TA Instruments Universal software version 4.5, under the expansion deformation mode, and using the adequate expansion probe, under ASTM E831–19 standard. TGA: TA Instruments Q500 analyzer using TA Instruments Universal software version 4.5. Flexural test: Tinius Olsen H25KS Universal Testing Machine, tree-point system according to ASTM D790–17. Literature survey for mechanical properties of commercial epoxy systems.
Data source location	The mechanical and thermal characterizations were performed at Escuela Politécnica Nacional (EPN), Quito, Ecuador*.*
Data accessibility	Repository name: Mendeley Data [1] Data identification number: 10.17632/bypjxzm3fn.1 Direct URL to data: https://data.mendeley.com/datasets/bypjxzm3fn/1
Related research article	None.

## 1 Value of the Data

• This dataset will serve as a tool for materials engineers for producing a tailor-made amine-epoxy system, based on its desired properties, through the selection of the post-curing temperature, which ultimately defines the crosslinking density of the system.
• Mechanical, civil, and materials engineers will benefit from this data by extending the glassy state of this amine-epoxy system through an adequate post-curing, since its elastic properties will remain stable up to elevated temperatures (at least 110 °C), which is crucial for high temperature applications in automotive, aeronautic, aerospace and construction.
• This dataset will aid materials engineers in achieving the maximum service temperature for this amine-epoxy system (120 °C), reported under unspecified post-curing conditions by the supplier as the heat deflection temperature (HDT).
• Industry will benefit from the considerably reduced post-curing time required by this structural amine-epoxy system (1–2 h), and the reproducible mechanical and thermal properties achieved in fully cured resins up to temperatures approaching T_g_∞, as demonstrated by this dataset.
• By adequately post-curing the epoxy, its curing kinetics would no longer manifest in thermal tests, allowing material scientists to evaluate its actual state by using the first cycle test run and eliminating the need to erase its thermal history beforehand.

## 2 Background

Post-curing is a necessary process for controlling the crosslinking in amine-hardened epoxy resins, especially when the initial curing is conducted at ambient temperatures [2]. When curing, epoxy resin reaches a glassy state before crosslinking is completed and thus the crosslinking process drastically diminishes as it is entirely dependent on diffusion mechanisms as opposed to the more-effective kinematic mechanisms that occur in the rubbery state. Therefore, exposing the resin to temperatures near its maximum glass transition temperature (T_g_∞) is required to promote its crosslinking and to reach a fully cured resin [3]. Subsequently, mechanical and thermal properties are directly related to the crosslinking degree [4,5]. In fact, since the glass transition temperature (T_g_) is a measurement of the polymer-chain mobility, it reaches its maximum when crosslinking is maximized, providing a valid tool for inferring the crosslinking degree of epoxy systems. Thus, this dataset was generated to characterize the Epon 828/Epikure 3223 epoxy system, as a function of three different post-curing temperatures (80, 100, 120 °C) to determine the variation of its mechanical and thermal properties. Besides, this work provides data supporting a post-curing procedure to extend the glassy region of this amine-epoxy system for achieving the maximum service temperature.

## 3 Data Description

The dataset is a compilation of the mechanical and thermal properties of a structural thermoset system, composed of an epoxy resin (Epon 828) and amine hardener (Epikure 3223), cured at room temperature (20 °C) and post-cured at 80, 100 or 120 °C. These post-curing temperatures were selected based on the indirect manufacturer recommendations and considering the literature precedent. First, the manufacturer states in the data sheet of EPON 828 that the epoxy system has a heat deflection temperature, i.e. temperature at which the polymer begins to deform, of 120 °C and thus was selected as the maximum post-curing temperature. On the other hand, the other post-curing temperatures were selected to compare our findings based on two different studies that reported mechanical properties of similar epoxy systems [6,7]. Furthermore, in a previous work we optimized our epoxy sample preparation procedure for a post-curing temperature of 80 °C, which served as basis for the methodology of this study [8]. Processed data on DSC, DMA, TMA, TGA, and flexural testing, along with statistical analysis of each of them are individually presented in this section.

### 3.1 DSC

**Fig. 1:**
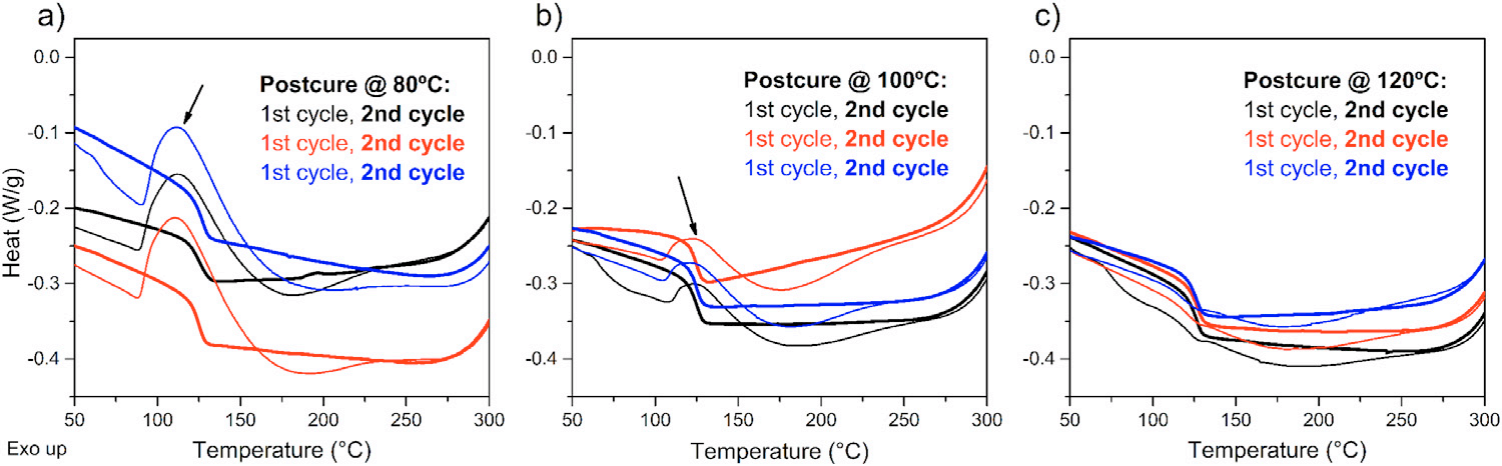
DSC curves for the first and second heating cycles of epoxy samples post-cured at a) 80, b) 100, and c) 120 °C. Red, blue and black represent each of the three samples tested for every post-curing temperature.

DSC tests were conducted to evaluate the curing state of the epoxy samples by the identification of exothermic peaks and the T_g_ region. For the uncured samples, these exothermic peaks are superimposed over the T_g_ step in the baseline of the curve. The DSC curves of epoxy samples post-cured at the three selected temperatures are shown in Fig. 1. Exothermic peaks, related to curing, appear solely in the first heating cycle for the samples post-cured at 80 and 100 °C, and are marked with arrows in Fig. 1, Fig. 1, respectively. The intensity of these peaks decreases as the post-curing temperature increases. Fig. 1c presents the DSC thermogram of specimens post-cured at 120 °C, which does not show an exothermic curing peak during the first and second cycles.

The T_g_ values of every epoxy sample post-cured at the three selected temperatures are summarized in Table 1, which were calculated from the second DSC cycle run. These T_g_ values were analyzed using one-way ANOVA. No statistically significant differences between T_g_ values of epoxy samples post-cured at 80, 100 and 120 °C were found, as the p-value= 0.07 is higher than 0.05.

**Table 1:** T_g_ obtained from the second cycle run of the DSC curves for epoxy samples exposed to the three post-curing temperatures.

Post-curing temperature (°C)	80	100	120
Tg* (°C)	125.10	125.13	126.29
	124.82	125.03	127.14
	126.98	124.74	126.58
Average	125.63	124.97	126.67
Standard deviation	1.17	0.20	0.43

⁎ Three samples were tested for each post-curing temperature.

### 3.2 DMA

**Fig. 2:**
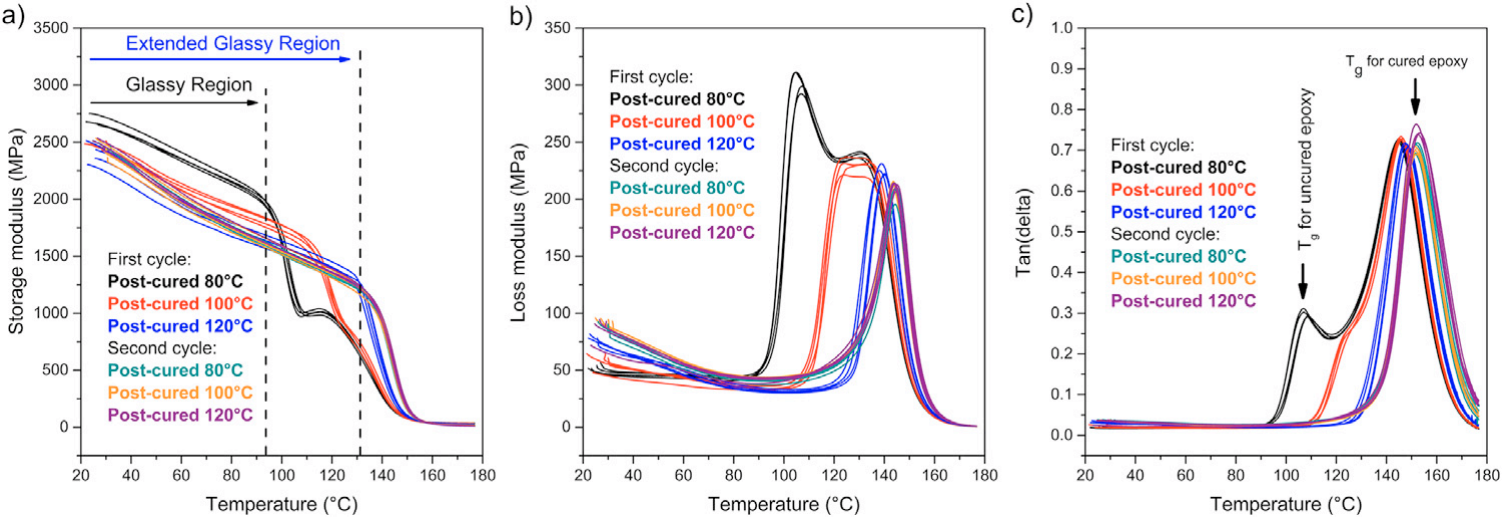
DMA curves, a) storage moduli, b) loss moduli and c) tan(delta) as a function of the post-curing temperature and the cycle run. Red, blue and black represent each of the four samples tested for every pos-curing temperature.

DMA tests were run to evaluate the differences in viscoelastic response of the post-cured samples as a function of temperature. Additionally, the curing state of the epoxy samples was evaluated regarding the Tan(delta) peaks, which reach a maximum when the curing is completed, and it occurs at a temperature that represents the T_g_. Fig. 2 presents the storage modulus, loss modulus and Tan(delta) curves of epoxy samples post-cured at 80, 100 and 120 °C, obtained from DMA, during the first and the second heating cycles. As the post-curing temperature increases, the storage modulus decreases, and the glassy region is extended (see Fig. 2a). Fig. 2b and Fig. 2c show that the loss modulus and the Tan(delta) vary for each post-curing temperature, respectively. During the first heating run, for the 80 °C post-curing temperature, the loss modulus curve is composed of two separate superimposed peaks: the first peak (∼105 °C) having a higher height than the second one (∼120 °C). On the contrary, the loss modulus curve presents two almost merged peaks at 125 – 135 °C for the 100 °C post-curing temperature, while this curve exhibited a single sharp peak at ∼147 °C for the 120 °C post-curing temperature. As for the second cycle, the loss modulus curves overlap in a single sharp peak (∼150 °C), independent of the post-curing temperature. The intensity of all loss modulus peaks decreases as post-curing temperature increases. The Tan(delta) peaks (see Fig. 2c) sharpen, and its height increases with the rise of the post-curing temperature. The maximum height of the peaks occurring at a temperature corresponding to the T_g_ for cured epoxy is achieved for the second cycle run of samples cured at 80, 100 and 120 °C. Only for sample cured at 120 °C, the T_g_ is already attained during the first heating cycle, as seen in Fig. 2c.

**Fig. 3:**
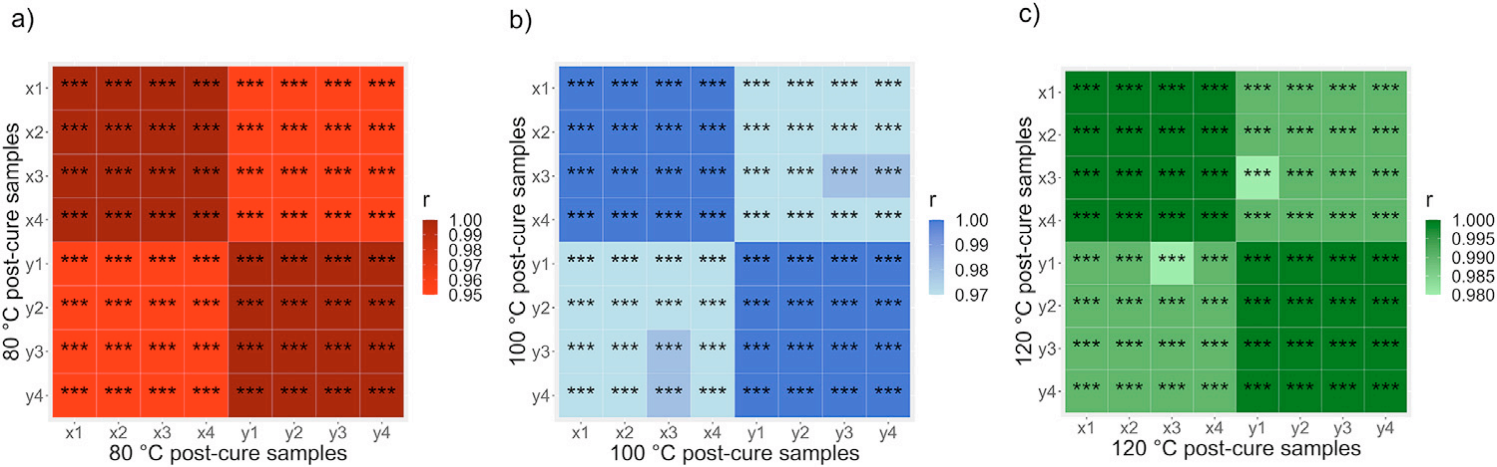
Pearson’s coefficient r heatmap matrix for the epoxy storage modulus for both cycle runs at post-curing temperatures of (a) 80, (b) 100, and (c) 120 °C. The four samples of each post-curing temperature are numbered following a letter “x” for the first cycle and a letter “y” for the second cycle.

The effects of the different post-curing temperatures on the storage modulus can be better appreciated in the correlation heatmap (Fig. 3) showing Pearson’s coefficient r for each compared curve, where the color scale indicates the correlation strength, and the statistical significance is represented by the p-value (*** means a p-value ≤ 0.001). This tool compares each data point of the DMA curve to determine existing relations between variables and quantifies the differences between the epoxy storage modulus, as a function of the post-curing temperature and the cycle run. Fig. 3a compares the two cycle runs of samples post-cured at 80 °C, where the correlation strength (0.95 to 1) can be appreciated to clearly differentiate the storage modulus from cycle 1 from cycle 2. Fig. 3b compares the two cycle runs of samples post-cured at 100 °C, where the correlation (0.97 to 1) is still enough to differentiate the storage modulus from cycle 1 and 2; and Fig. 3c compares the two runs of samples post-cured at 120 °C, where the correlation (0.98 to 1) can vaguely differentiate the storage modulus from cycle 1 and cycle 2. Thus, the correlation between the storage modulus measured during the first and second cycles increases as the post-cure temperature increases. At a post-curing temperature of 120 °C, the storage modulus values are statistically equal between the first and second cycle.

**Fig. 4:**
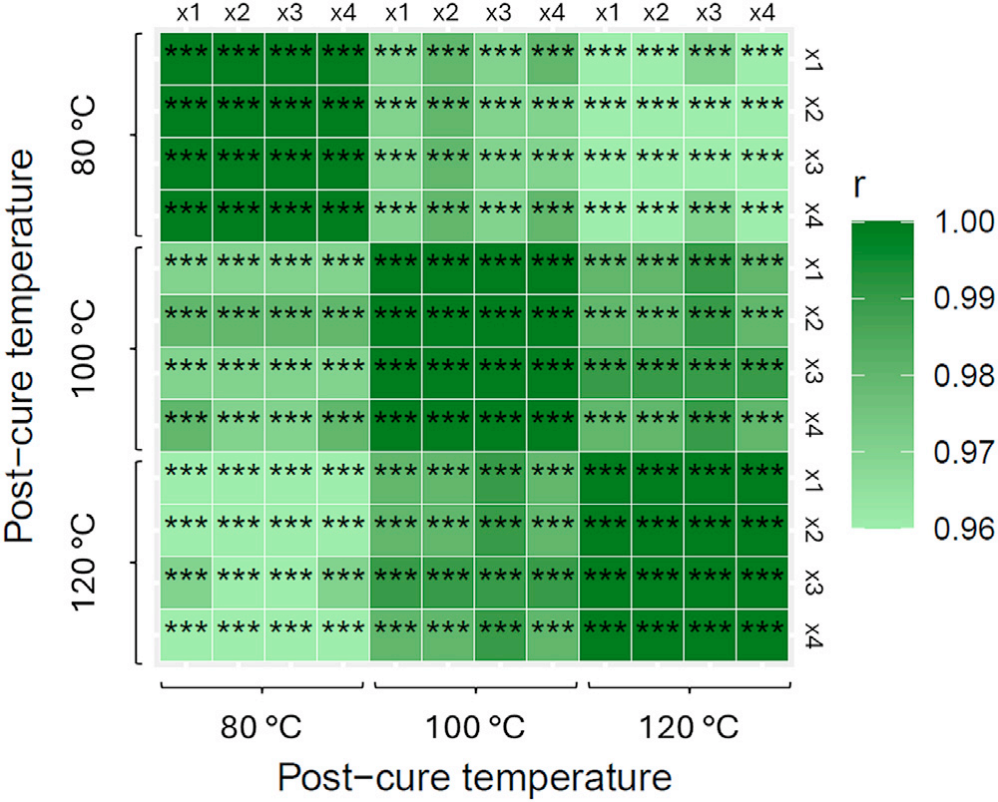
Pearson’s coefficient r heatmap matrix for the first cycle run of epoxy storage modulus. The four samples of each post-curing temperature are numbered following a letter “x” for the first cycle.

**Fig. 5:**
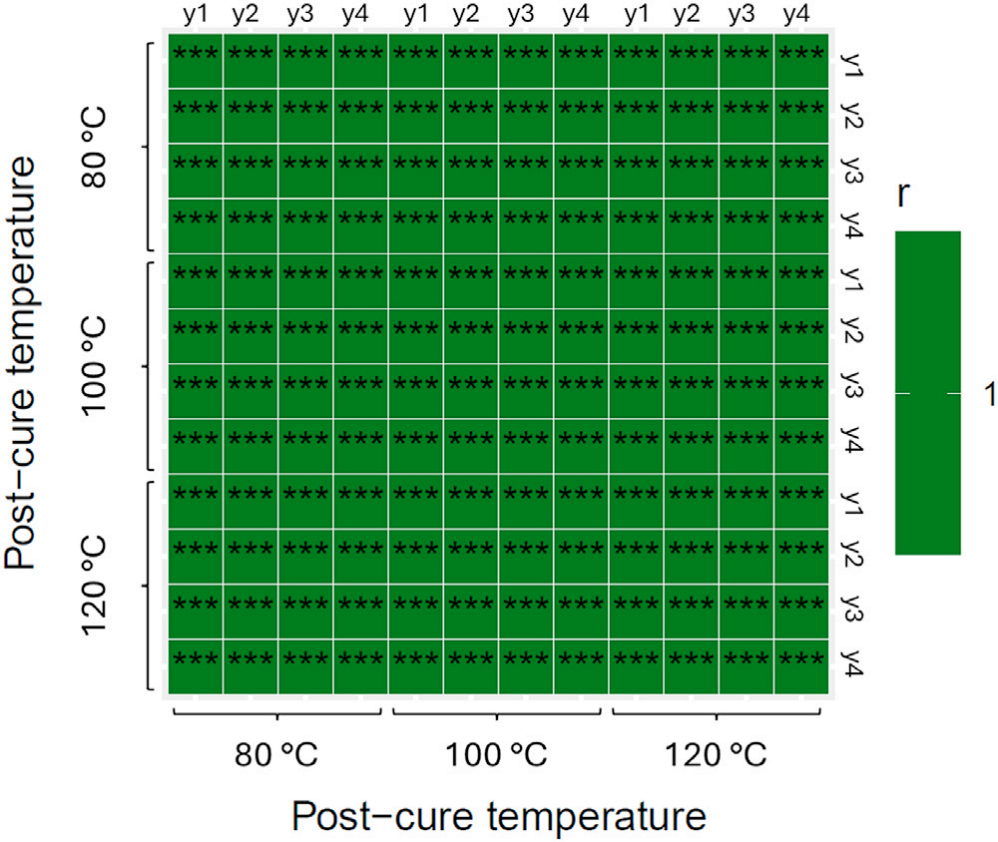
Pearson’s coefficient r heatmap matrix for the second cycle run of epoxy storage modulus. The four samples of each post-curing temperature are numbered following a letter “y” for the second cycle.

The epoxy storage modulus data for the first and second cycles, across all post-curing temperatures, are presented in Fig. 4, Fig. 5, respectively. In Fig. 4, color variations clearly indicate differences related to post-curing temperatures. In contrast, Fig. 5 shows uniform color across all temperatures, suggesting consistent storage modulus values after the second cycle, independent of the post-curing temperature. The correlation strength of the storage modulus curves shown in both figures have a high statistical significance.

Additionally, the storage modulus during the first cycle run was computed at five potential service temperatures (40, 60, 80, 100, and 120 °C) within the glassy region of the DMA curves presented in Fig. 2a, for all post-curing temperatures. This analysis evaluated the temperature-dependent elastic behavior of the epoxy samples (Table 2). Note that calculations were unfeasible at 100 °C for samples post-cured at 80 °C, and at 120 °C for samples post-cured at 80 and 100 °C, as these service temperatures coincided with the transition from the glassy to the rubbery state occurring for those samples. The storage modulus values were analyzed through Two-way ANOVA that determined statistically significant differences amongst the three selected temperatures (p-value=6.93E-24) and the post-curing temperatures (p-value=2.19E-21), as well as interactions between the temperature at which the storage modulus was calculated and the post-curing temperature (p-value=1.51E-05).

**Table 2:** Storage modulus computed at 40, 60, 80,100, and 120 °C within the glassy region of the DMA curves for the first cycle run.

		Storage modulus (MPa)				
	ST*	@ 40 (°C)	@ 60 (°C)	@ 80 (°C)	@ 100 (°C)	@ 120 (°C)
Post-curing temperature (°C)	80	2572.82 ± 36.70	2363.75 ± 33.33	2155.67 ± 27.62	⁎⁎	⁎⁎
	100	2343.10 ± 27.87	2101.66 ± 28.68	1914.60 ± 37.08	1740.95 ± 38.86	⁎⁎
	120	2300.62 ± 31.16	1940.24 ± 34.40	1701.81 ± 28.25	1528.66 ± 25.82	1330.07 ± 20.40

⁎ Service temperature.

⁎⁎ The storage modulus is not calculated due to the glassy-to-rubbery transition.

The T_g_ values were also computed through the Tan(delta) peaks (see Table 3) from DMA curves exhibited at Fig. 2c. For the first cycle run, the T_g_ increases as the post-curing temperature increases, while for the second cycle run, the T_g_ reaches its maximum value of ∼153 °C, which practically corresponds to T_g_∞ for this amine-epoxy system. Two-way ANOVA was used to simultaneously analyze the effects of both factors (post-curing temperature and cycle run) on T_g_. It was determined that this property is dependent on both factors as T_g_ values present statistically significant differences between cycle runs (p-value=1.3E-18) and between post-curing temperatures (p-value=2.2E-7). Furthermore, there is a significant interaction between both factors (post-curing temperature and cycle runs) based on the p-value=0.03, suggesting that the factors are dependent on each other.

**Table 3:** T_g_ values determined by DMA Tan(delta).

Post-curing temperature (°C)	80		100		120	
Cycle run	1	2	1	2	1	2
Glass transition temperature (°C)	144.46	151.68	146.53	152.07	147.70	153.21
	145.12	152.26	145.75	151.55	147.05	153.32
	144.75	151.64	145.65	151.46	146.37	152.48
	144.99	151.42	145.40	151.44	147.03	153.13
Average	144.83	151.75	145.83	151.63	147.04	153.04
Standard deviation	0.29	0.36	0.49	0.30	0.54	0.38

### 3.3 TMA

**Fig. 6:**
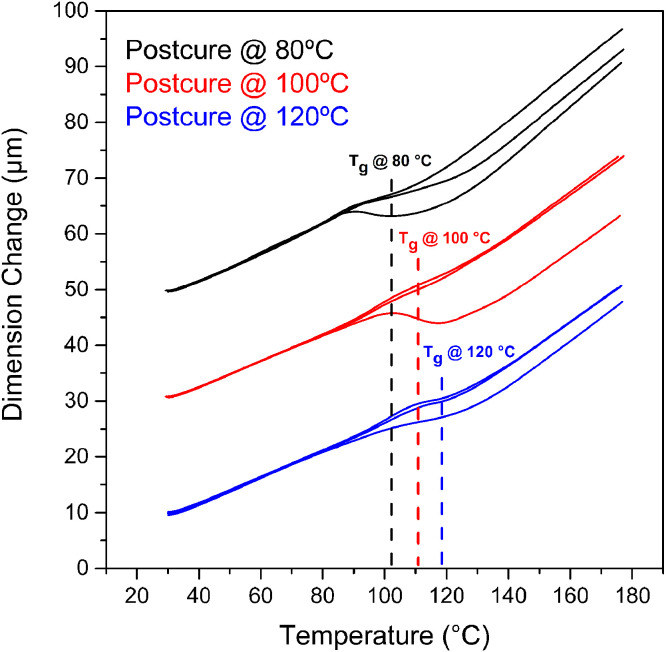
TMA curves of epoxy samples (first heating run) for each post-curing treatment. Red, blue and black represent each of the three samples tested for every pos-curing temperature.

The TMA curves during the first heating cycle are presented in Fig. 6 and analyzed to obtain the coefficient of thermal expansion (CTE) of epoxy samples for both glassy and rubbery regions, shown in Table 4, respectively. One-way ANOVA was used to determine statistically significant differences between CTEs, showing that the glassy CTE (p-value=0.36) were constant while the rubbery CTE (p-value=0.02) has significant differences amongst post-curing temperatures. Also, the T_g_ increases as a function of the post-curing temperatures.

**Table 4:** CTE of epoxy samples for the first heating run, in the glassy and rubbery regions of TMA curves.

Region	Glassy			Rubbery		
Post-curing temperature (°C)	80	100	120	80	100	120
CTE (µm/m°C)	80.74	78.45	77.12	141.33	124.36	127.66
	80.50	82.29	80.42	147.91	135.61	127.42
	81.82	78.11	79.15	138.83	128.67	131.93
Average	81.02	79.62	78.90	142.69	129.55	129.00
Standard Deviation	0.70	2.32	1.66	4.69	5.68	2.54

### 3.4 TGA

**Fig. 7:**
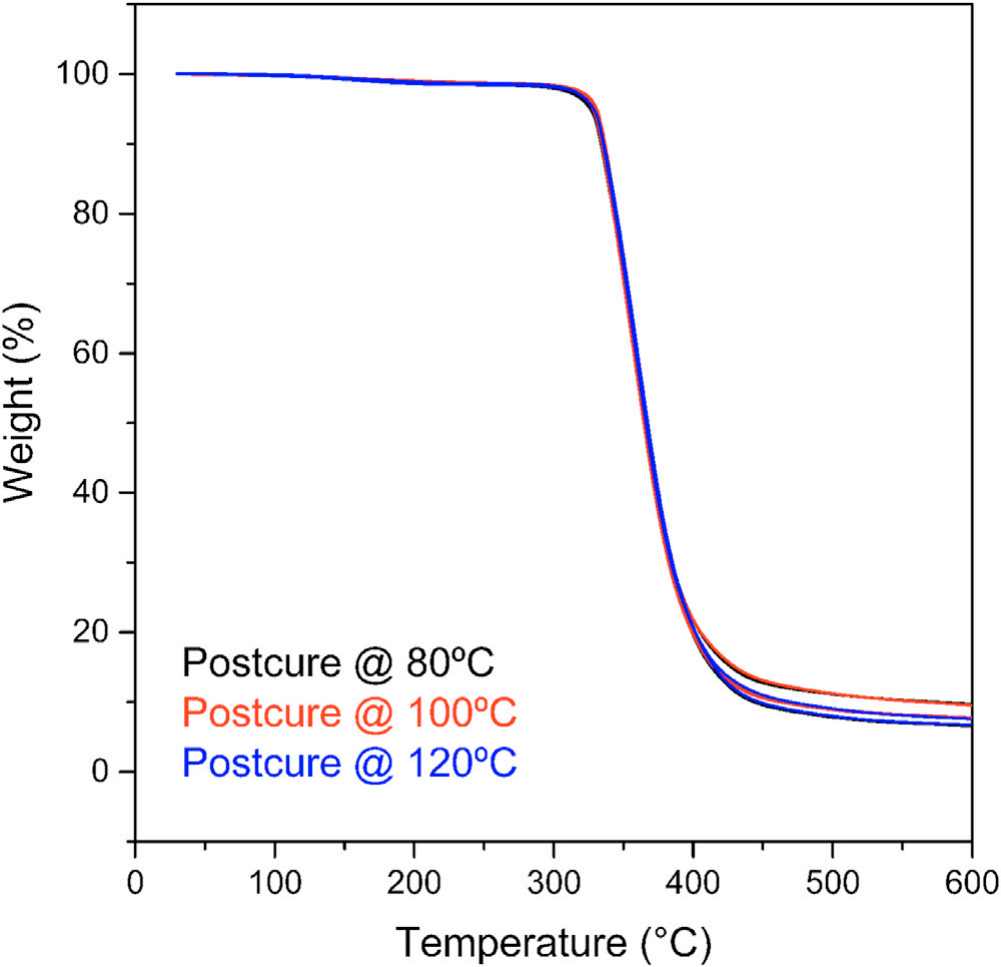
TGA curves during the first heating run of epoxy samples post-cured at the selected temperatures. Red, blue and black represent each of the three samples tested for every post-curing temperature.

Finally, TGA was carried out to identify the mass loss of the samples as a function of temperature. The TGA results of epoxy samples for the first heating cycle are plotted in Fig. 7, showing consistency in the thermal degradation amongst the post-curing temperatures.

### 3.5 Flexural tests

**Fig. 8:**
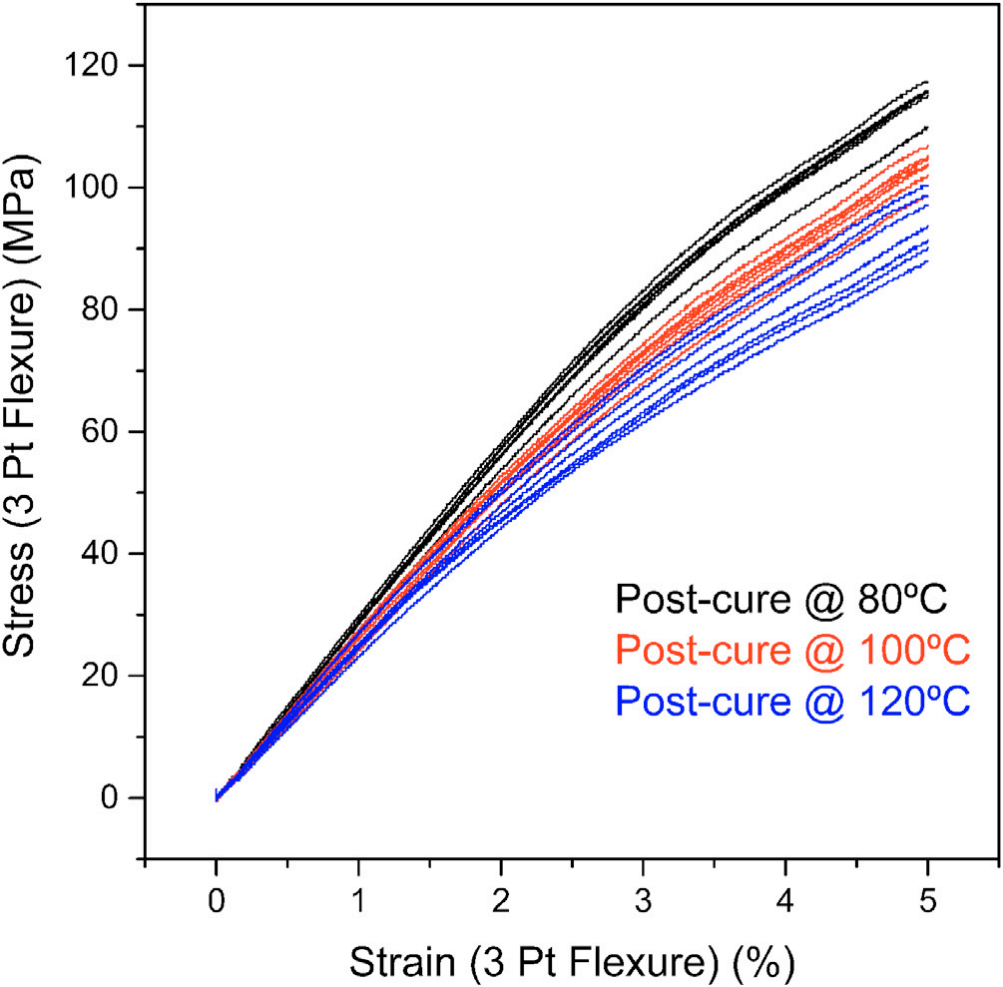
Flexural stress-strain curves for epoxy samples exposed to different post-curing temperatures. Red, blue and black represent each of the seven specimens tested for every post-curing temperature.

**Fig. 9:**
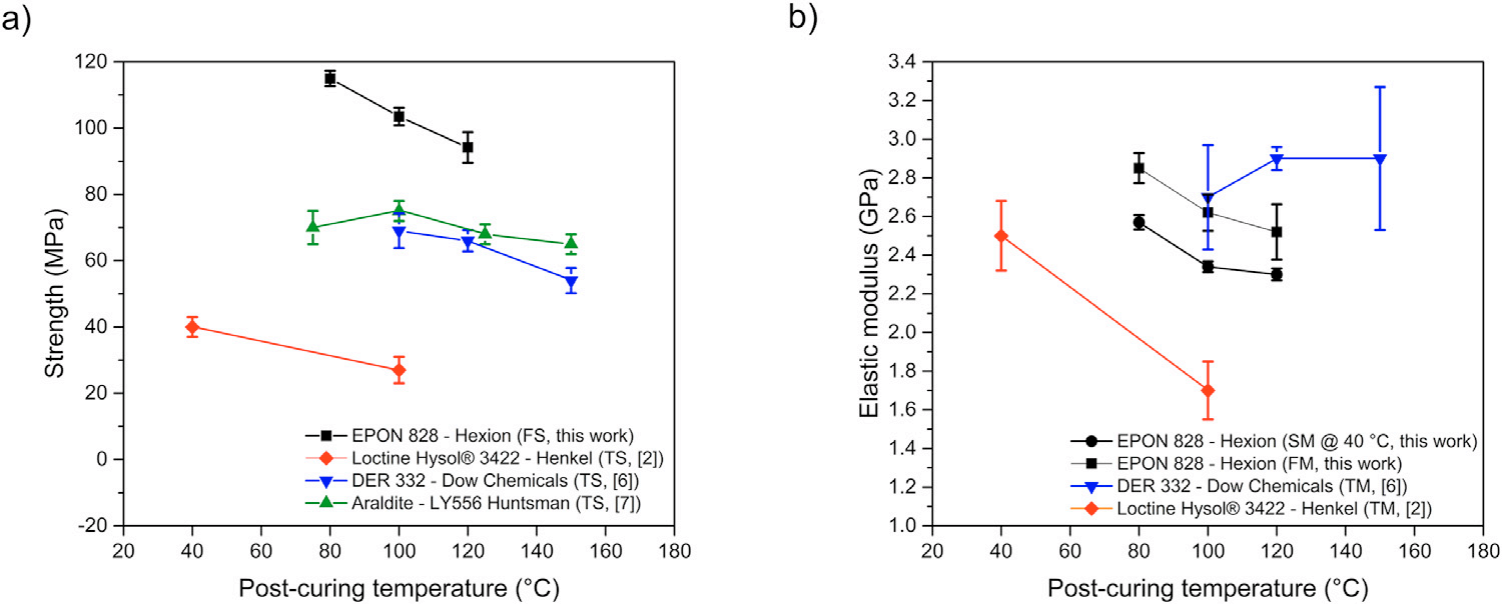
(a) Strength and (b) elastic modulus of the EPON 828 epoxy system studied in this work, plotted as a function of post-curing temperature and compared with other commercial epoxy-amine systems. FS: flexural strength, TS: tensile strength, FM: flexural modulus, TM: tensile modulus and SM: storage modulus.

The flexural stress-strain curves of epoxy samples are shown in Fig. 8, while the values of the corresponding properties obtained from them are summarized in Table 5. The flexural modulus and strength were averaged from the flexural stress-stain curves and plotted with their corresponding standard deviations, as seen in Fig. 9. ANOVA analysis determined that the flexural strength (p-value=4.29E-09) and moduli (p-value=6.84E-05) had statistically significant differences when compared for the different post-curing temperatures. The results demonstrate that the flexural strength and flexural modulus decrease as the post-curing temperature increases, following the trend observed in Fig. 2a for the storage modulus.

**Table 5:** Flexural properties summary for epoxy samples post-cured at different temperatures.

Post-curing temperature (°C)	Flexural strength (MPa)	Deformation (%)	Flexural modulus (MPa)
80	114.99 ± 2.33	5.00	2854.14 ± 77.88
100	103.50 ± 2.66	5.00	2621.37 ± 94.47
120	94.15 ± 4.62	5.00	2521.76 ± 143.86

Fig. 9 compares the flexural properties and storage modulus trends reported in this work with literature data for commercial epoxy/amine systems. These systems, cured at ambient temperature and post-cured at various temperatures, demonstrate consistent mechanical property trends.

### 3.6 Summary

**Fig. 10:**
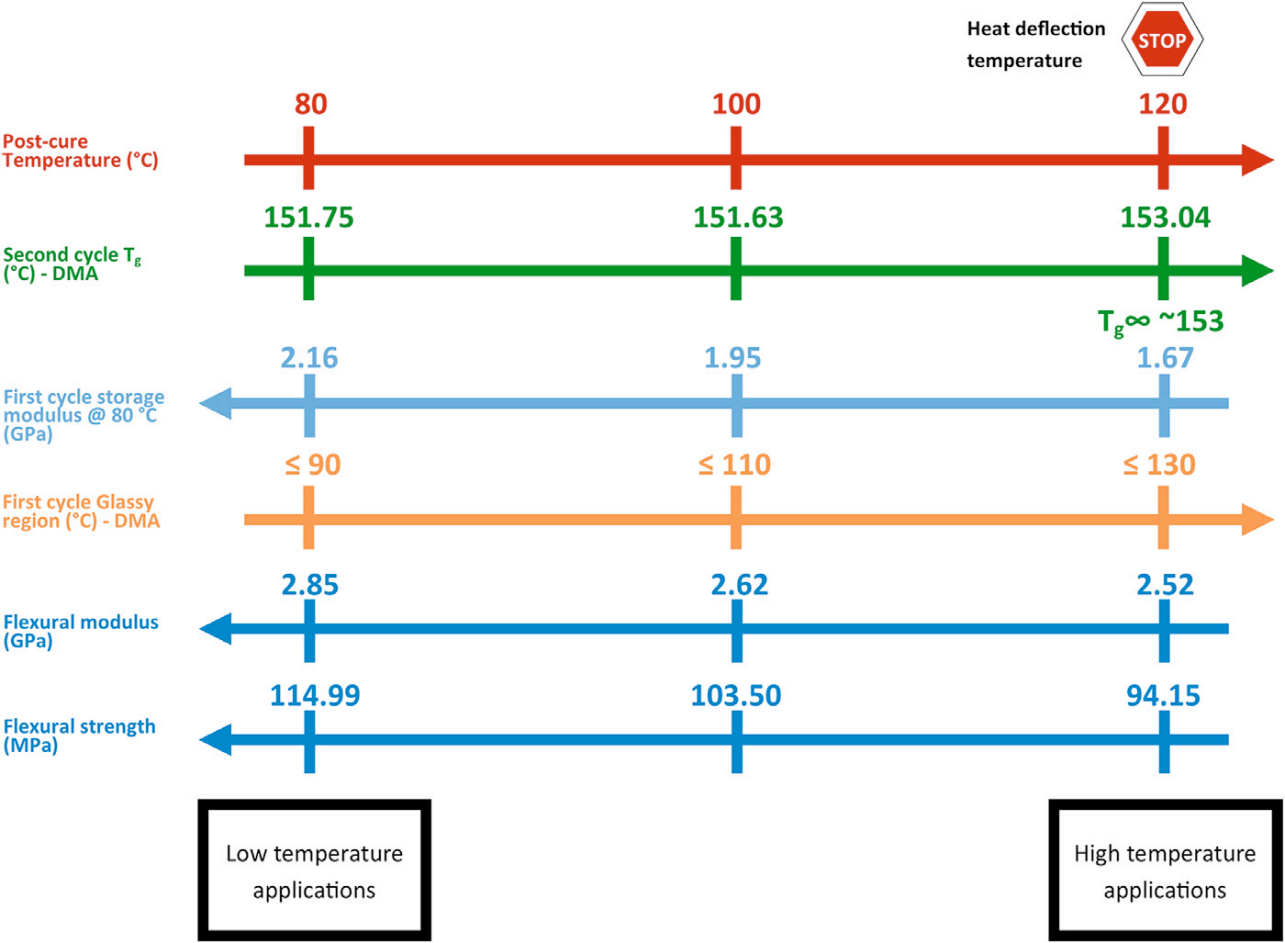
Data summarizing the influence of post-curing temperature in the glassy temperature range, storage modulus at service temperature of 80 °C, flexural strength, and flexural modulus of the Epon 828/Epikure 3223 amine-epoxy system, cured at ambient temperature.

The data obtained from this article can be summarized in Fig. 10.

## 4 Experimental Design, Materials and Methods

### 4.1 Material selection

Epoxy system was composed of a bifunctional resin derived from diglycidyl ether of bisphenol A (DGEBA), with the commercial brand name of Epon 828, and a multifunctional amine resin hardener derived from diethylenetriamine, with the commercial brand name of Epikure 3223. The mass ratio between EPON 828 and EPIKURE 3223 was 100:12 and the materials were supplied by Hexion Inc., Columbus Ohio. The service temperature for Epon 828/Epikure 3223 system was defined by the supplier as being 120 °C in terms of the heat deflection temperature (HDT) for curing at room temperature and post-curing at elevated temperature (non-specified in the data sheet). The time for curing and post-curing this amine-epoxy system to achieve the HDT is also not reported by the supplier.

### 4.2 Sample preparation

**Fig. 11:**
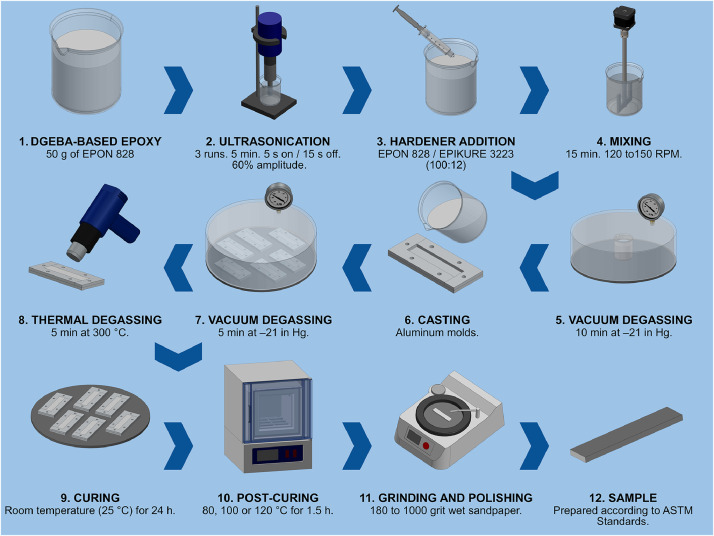
Procedure for the preparation of epoxy samples.

The epoxy samples were prepared based on the process detailed in Fig. 11. Initially, the liquid epoxy resin was sonicated by an ultrasonic probe to break-up semicrystalline structures formed during storage. The hardener was then added to the resin and thoroughly mixed by a mechanical agitator to ensure homogeneity. Immediately after, the mixture was poured into aluminum molds, degasified in a vacuum chamber, and briefly heated with a heat gun to eliminate air bubbles trapped in the surface. The samples were then left to cure at room temperature for 24 h before unmolding and subjecting them to their corresponding post-curing temperature (80, 100 and 120 °C) for 1.5 h. Room temperature curing was selected to limit warping and porosity of the thermoset specimens, after which post-curing is necessary to increase crosslinking degree. The amine/epoxy system requires an initial curing at room temperature, so the polymer chains have sufficient time for crosslinking to occur. However, since this curing process is an exothermic reaction, applying additional heat to the system would cause abrupt crosslinking. As a result, warping and porosity is often present in epoxy resin that has been initially cured at higher temperatures [3].

### 4.3 Software

Most figures were plotted using Origin Pro 2016 (Version b9.3.226) 64-bit and Adobe Photoshop (Version 21.0.1) 64-bit. The Pearson’s coefficient r heatmap matrix was calculated and plotted using RStudio version 2023.06.0 Build 421 using the rstatix and ggplot2 libraries, respectively. ANOVA analysis was performed using the Data Analysis toolbox in Microsoft Excel for Microsoft 365 MSO (Version 2412 Build 16.0.18324.20092) 64-bit. The schematics were made using Publisher for Microsoft 365 MSO (Version 2505 Build 16.0.18827.20102) 64-bit.

### 4.4 Mechanical tests

*DMA:* A 35 mm dual-cantilever clamp apparatus, suitable for beam flexure operation, was used. A temperature ramp oscillation test mode, that applies a sinusoidal load to the sample and measures its deformation as a function of temperature, was used under the following settings: four samples of 12.7 × 3.2 × 62 mm, 30 µm amplitude, 1 Hz frequency, 3 °C / min ramp rate, 30 to 180 °C temperature range, two consecutive cycle runs. The test was carried out in a TA Instruments Discovery DMA 850 analyzer, using TRIOS software version 5.0 in compliance with ASTM D5418 [9] and ASTM E1640–18 [10] standards. The storage modulus, loss modulus and Tan (delta) were obtained through DMA curves for the four samples post-cured at the selected temperatures. Additionally, the storage modulus was statistically analyzed at 40, 60, 80, 100 and 120 °C, defined as potential service temperatures within the glassy region of the DMA curve, to evenly evaluate the temperature effects on the elasticity of the composites.

*Flexural test:* Seven specimens (3.2 × 12.7 × 64 mm) for each post-curing temperature were tested using a 16:1 ratio of support span and specimen depth. The specimen has an overhang of 6.4 mm on each end of the support span (51.2 mm) to prevent slipping. The rate of crosshead motion was determined as ∼1.37 mm/min, calculated using a straining rate of 0.01 (mm/mm)/min as indicated in the test standard. The test was carried out using a Tinius Olsen H25KS universal testing machine, three-point system according to ASTM D790–17 [11]. The flexural modulus of elasticity and the flexural strength were calculated from the measured force and deflection, based on the calculation procedure specified in the test standard. The arithmetic mean of the data values were reported as the average for the assessed properties.

### 4.5 Thermal tests

TMA: Three epoxy samples of 3 × 3 × 3 mm were tested for each post-curing temperature. Temperature ramp method with expansion probe was used under the following conditions: 50 mL / min nitrogen atmosphere, 30 to 180 °C temperature range, 3 °C / min ramp rate, 0.1 N initial force, 0.05 N incremental force. The linear coefficient of thermal expansion is calculated as a function of the slope corresponding to a temperature range from a linear portion of the thermomechanical curve, as specified in the ASTM E831–19 standard [12]. The tests were carried out in a TA Instruments Q400 analyzer using TA Instruments Universal software version 4.5, under the expansion deformation mode, and using the adequate expansion probe.

*DSC:* Three epoxy samples for each post-curing temperature were prepared in the following dimensions: 4 mm diameter and 1 mm thick. Each sample was tested in aluminum pans according to the following settings: two heating cycles in a temperature range of 30 to 300 °C separated by an equilibrium-cooling, a nitrogen atmosphere flow rate of 15 mL / min, and a heating ramp of 10 °C / min. The test were performed in a TA Instruments Q2000 analyzer using TA Instruments Universal software version 4.5, under the procedure indicated in the ASTM E2602–22 standard [13].

*TGA:* Three epoxy samples (∼10 mg each) for each post-curing temperature were prepared and tested under the following test conditions: a nitrogen atmosphere, a 20 mL / min microbalance flow-rate, an 80 mL / min sample flow-rate, a temperature range from 30 to 600 °C, and a heating ramp of 10 °C / min. The tests were performed in a TA Instruments Q500 analyzer using TA Instruments Universal software version 4.5.

**Fig. 12:**
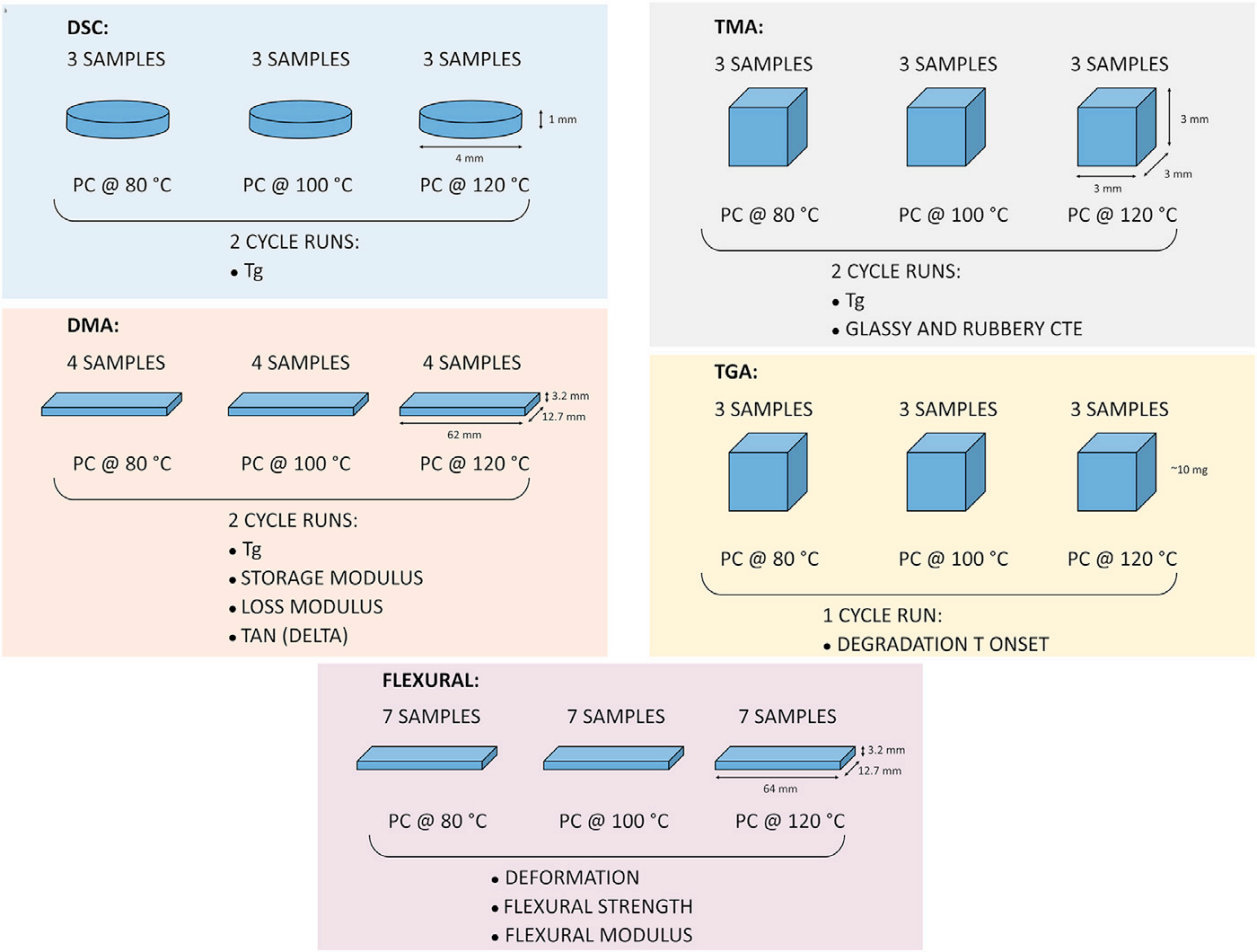
Schematic of mechanical and thermal characterization. Number of samples, sample dimensions and number of thermal cycles are included.

Fig. 12 presents a scheme of the mechanical and thermal characterization, including the number of samples, dimensions and heating cycles, as well as the measured or calculated properties.

## Limitations

None.

## Ethics Statement

We, the authors, hereby confirm that we have diligently reviewed and complied with all the ethical requirements for publication in Data in Brief. Furthermore, we affirm that the current work does not involve human subjects, animal experiments, or any data collected from social media platforms. Our research adheres to the ethical guidelines and principles set forth by the journal, and we have taken all necessary steps to ensure the responsible and ethical conduct of our research.

## CRediT Author Statement

**Alex Tamayo-Aguilar:** Conceptualization, Validation, Writing –original draft; **Víctor H. Guerrero:** Formal analysis, Writing –review & editing; Conceptualization, Supervision, Formal analysis; **Patricia I. Pontón:** Methodology, Data curation, Writing –review & editing; **Marco V. Guamán:** Conceptualization, Supervision, Formal analysis, Writing –review & editing.

## Declaration of Competing Interest

The authors declare that they have no known competing financial interests or personal relationships that could have appeared to influence the work reported in this paper.

## Data Availability

Mendeley DataData on mechanical and thermal properties of an amine-epoxy system at various post-curing temperatures (Original data).
